# Quantification of nucleic acid quality in postmortem tissues from a cancer research autopsy program

**DOI:** 10.18632/oncotarget.11836

**Published:** 2016-09-02

**Authors:** Jun Fan, Raya Khanin, Hitomi Sakamoto, Yi Zhong, Chelsea Michael, Derwin Pena, Breanna Javier, Laura D. Wood, Christine A. Iacobuzio-Donahue

**Affiliations:** ^1^ Sloan Kettering Institute, Memorial Sloan Kettering Cancer Center, New York, NY, 10065, USA; ^2^ Bioinformatics Core, Memorial Sloan Kettering Cancer Center, New York, NY, 10065, USA; ^3^ Department of Pathology, Memorial Sloan Kettering Cancer Center, New York, NY, 10065, USA; ^4^ Human Oncology and Pathogenesis Program, Memorial Sloan Kettering Cancer Center, New York, NY, 10065, USA; ^5^ David M. Rubenstein Center for Pancreatic Cancer Research, Memorial Sloan Kettering Cancer Center, New York, NY, 10065, USA; ^6^ Department of Pathology, Sol Goldman Pancreatic Cancer Research Center, Johns Hopkins University School of Medicine, Baltimore, MD, 21231, USA

**Keywords:** autopsy, RNA, post-mortem, RNA sequencing, metastasis

## Abstract

The last decade has seen a marked rise in the use of cancer tissues obtained from research autopsies. Such resources have been invaluable for studying cancer evolution or the mechanisms of therapeutic resistance to targeted therapies. Degradation of biomolecules is a potential challenge to usage of cancer tissues obtained in the post-mortem setting and remains incompletely studied. We analysed the nucleic acid quality in 371 different frozen tissue samples collected from 80 patients who underwent a research autopsy, including eight normal tissue types, primary and metastatic tumors. Our results indicate that RNA integrity number (RIN) of normal tissues decline with the elongation of post-mortem interval (PMI) in a tissue-type specific manner. Unlike normal tissues, the RNA quality of cancer tissues is highly variable with respect to post-mortem interval. The kinetics of DNA damage also has tissue type-specific features. Moreover, while DNA degradation is an indicator of low RNA quality, the converse is not true. Finally, we show that despite RIN values as low as 5.0, robust data can be obtained by RNA sequencing that reliably discriminates expression signatures.

## INTRODUCTION

Autopsy, derived from the Greek word *autopsia* meaning “to see for oneself”, is a method used since the 17^th^ century to learn about disease and determine the cause of death [1]. Autopsy was a main form of understanding disease until the mid-20^th^ century when medical imaging developed and allowed a view of the internal organs in a living patient [2]. In turn, the growth of laboratory medicine further diminished the need for autopsy as a diagnostic tool [3]. While postmortem exam has remained fundamental to improving knowledge of brain diseases, particularly neurodegenerative disorders, a renewed interest in its use for studying human cancer has gained traction in the past decade [4].

Sequencing of the human genome has led to a revolution in understanding of cancer etiology by revealing the genetic alterations characteristic of human tumors [5–7], the genetic features that underlie subtypes within a primary tumor type [8–11], or the mechanisms of therapeutic resistance [12–14]. With these advances has come a revival of interest in postmortem tissue collection because advanced stage disease is typically not accessible for study by next-generation methods in samples from living patients. As a result, research autopsy programs have emerged as a critical tool towards understanding the biology of lethal cancer and in many instances have led to significant insights into cancer progression and treatment resistance not possible with small tumor biopsies [14–16].

Despite the emergence of and implementation of research autopsy programs at a variety of institutions for obtaining cancer tissues, to date there are few formal evaluations of the quality of biomolecules in postmortem materials. A challenge to performing such evaluations is limited tissue resources. In some instances carefully screened cases and selected tissue types have been used to establish the relationship between potential quality-controlling factors and tissue sample quality [17–20]. Alternatively, simulated postmortem scenarios are used to mimic the postmortem interval and natural environment [21, 22]. Therefore, towards the goal of fully understanding these issues we leveraged our experience and resources amassed while running a cancer research autopsy program spanning more than a decade to determine the quality of nucleic acids in relation to tissue of origin, postmortem interval, normal versus neoplastic histology, primary versus metastases, and performance in downstream next generation sequencing methodologies.

## RESULTS AND DISCUSSION

### Sample set characteristics

**Figure 1:**
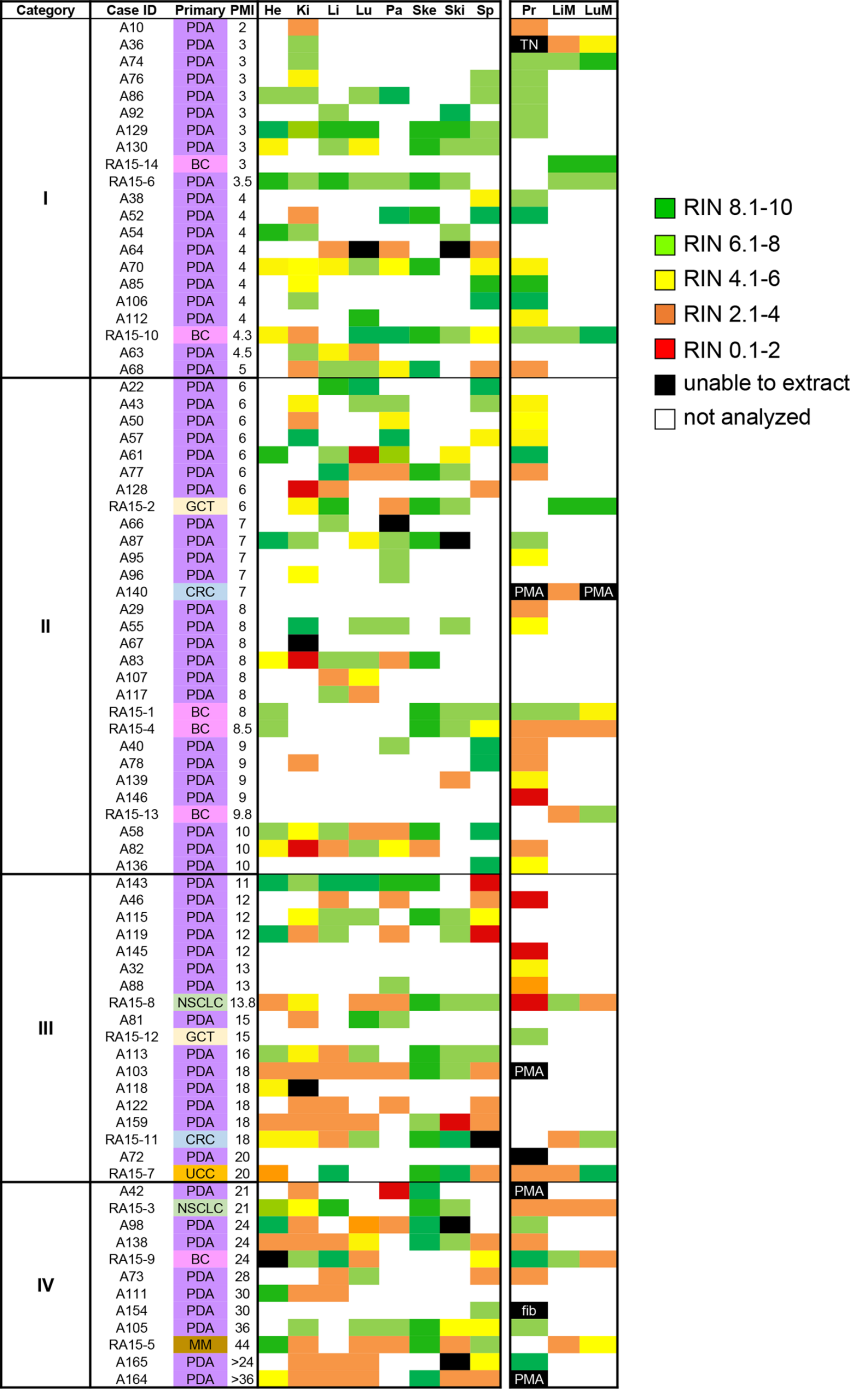
Heat map of RNA integrity numbers by tissue type RIN values from individual tissues were expressed with colored regions as defined by the accompanying legend. Abbreviations are PDA, pancreatic ductal adenocarcinoma; BC, breast cancer; GCT, germ cell tumor; CRC, colorectal cancer; NSCLC, non-small cell lung cancer; UCC, urothelial cell carcinoma; MM, melanoma; He, heart; Ki, kidney; Li, liver; Lu, Lung; Pa, pancreas; Ski, skin; Sp, spleen; Pr, primary tumor; LiM, liver metastasis; LuM, Lung metastasis. PMA, postmortem autolysis; TN, tumor-associated necrosis; fib, fibrosis.

Nucleic acid quality was analyzed in 371 different frozen tissue samples collected from 80 autopsied patients, 81% of which had been diagnosed with pancreatic cancer. The remaining patients had been diagnosed with breast cancer, lung cancer, colorectal cancer, germ cell tumor or melanoma (Figure 1). The postmortem interval (PMI) of these 80 autopsies ranged from 2 hours to > 36 hours. Cases with short PMIs were typically for those patients who expired while at the hospital, whereas cases with very long PMIs were a result of many factors including transport from outside the hospital or consent in the postmortem period by the patients' legally authorized representative to the program. Among the 371 tissue samples, 287 were histologically confirmed normal tissues sampled from the liver, lung, kidney, pancreas, spleen, heart, skeletal muscle and skin with a median of 35 normal tissues per site (range 30 to 49). We also collected 84 tumor samples of which 52 were from primary tumors, 16 from liver metastases and 16 from lung metastases including 10 patient-matched primary-metastatic pairs. To facilitate analyses, samples were arbitrarily categorized into four groups based on PMI: Category I, PMI ≤ 5 hours; Category II, PMI 6–10 hours; Category III, PMI 11–20 hours; and Category IV, ≥ 21 hours. When tumor samples were included there were a total of 98 samples from PMI Category I, 110 samples from PMI Category II, 88 samples from Category III, and 74 samples from Category IV (Table 1).

**Table 1:** Sample cohort

Postmortem Interval	Heart	Kidney	Liver	Lung	Pancreas	Skeletal Muscle	Skin	Spleen	Primary Tumor	Liver Metastases	Lung Metastases
Category I (1–5 h)	7	15	8	10	7	7	7	12	15	5	5
Category II (6–10 h)	7	13	11	11	15	8	8	10	18	5	5
Category III (11–20 h)	9	11	10	8	8	8	8	11	9	3	3
Category IV (> 21 h)	7	10	7	8	4	7	7	8	10	3	3
Total Samples	30	49	36	37	34	30	30	41	52	16	16

### RNA integrity in normal tissues is tissue-type specific

We first determined the extent to which RNA could be extracted from this large set of postmortem normal tissues. RNA was successfully extracted from 269 of 287 (94%) samples attempted. The overall average RIN for all 269 samples was 5.94 ± 2.5, with a median RIN of 6.4. By contrast, for 10 of 287 samples (6%) the RNA yield was exceedingly low leading to unreported or unreliable RIN, and these samples were assigned an RIN value of 0 (Figure 1). In general, higher overall RNA yields were found from tissue samples collected within 1 year compared to those with long-term storage (> 5 years). This was unrelated to the number of freeze/thaw cycles per sample as with rare exceptions all frozen normals analyzed were previously unused and continuously stored at −80°C. Furthermore, low RNA yields (defined as 20 ug/ml total RNA) were unrelated to PMI nor were they related to a specific histology.

**Figure 2:**
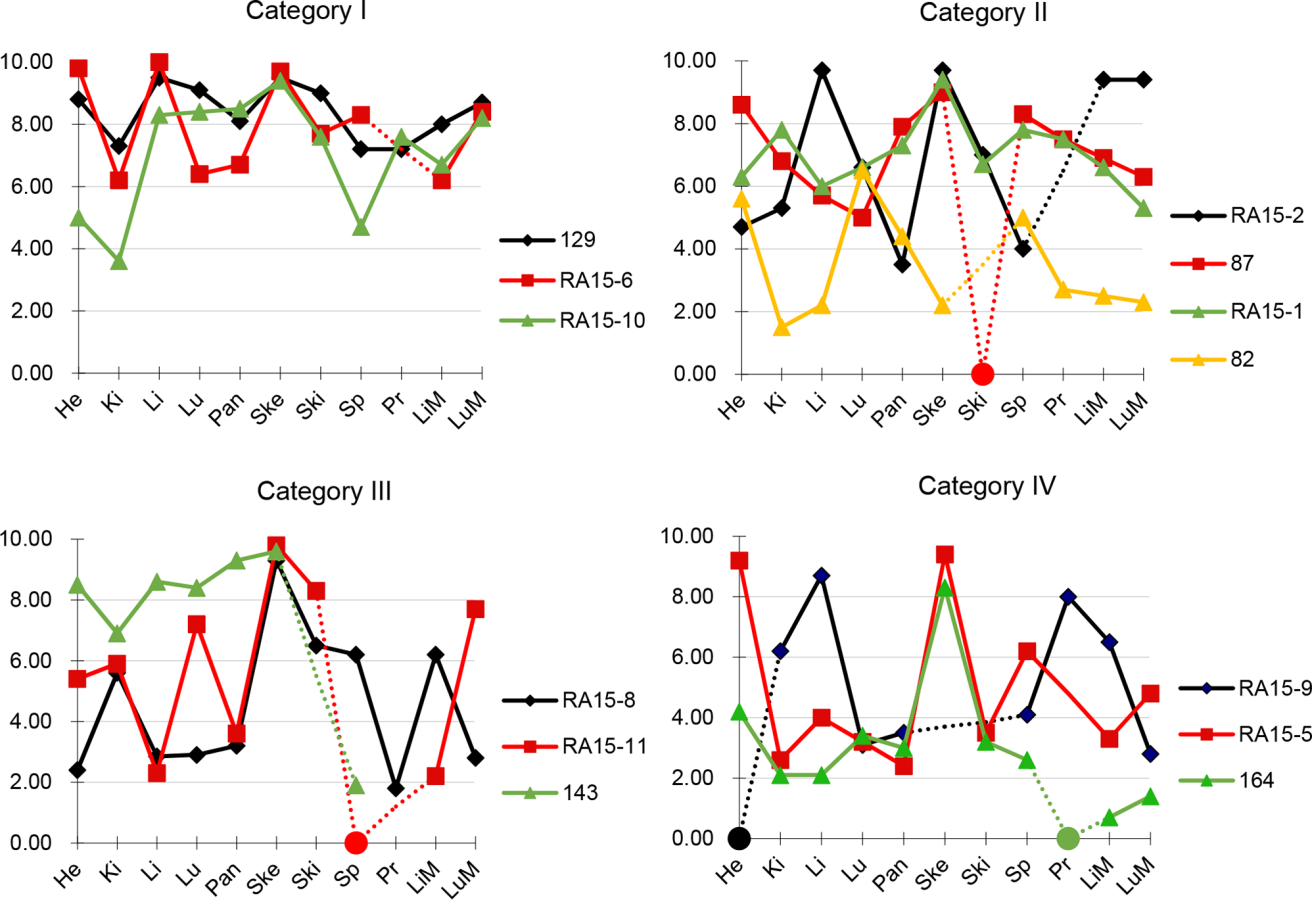
RNA integrity numbers in patient-matched tissues RIN values from patient-matched tissues were plotted against tissue types. Selected cases from each of the four PMI categories were shown. Samples with low RNA yield leading to unreported or unreliable RIN were assigned to a RIN value of 0 and indicated by a circle. Dashed lines indicate tissues not analysed for that patient. Abbreviations are He, heart; Ki, kidney; Li, liver; Lu, Lung; Pa, pancreas; Ske, skeletal muscle; Ski, skin; Sp, spleen; Pr, primary tumor; LiM, liver mets; LuM, Lung mets.

We next calculated the mean RIN values for each individual tissue type for which RNA was obtained (Table 2). Overall, mean RIN values showed little variability among the eight normal tissue types and most tissue types had RIN values between 5 to 6.5. The tissue with the lowest mean RIN value was the kidney (RIN 4.63 ± 1.95) whereas the highest values were noted for skeletal muscle (RIN 9.01 ± 1.36), suggesting tissue-type specific differences in RNA stability in the postmortem interval. This pattern did not change when the median RIN value in each category was alternatively considered. To determine the extent to which RIN values of normal tissues show intra-patient variability we evaluated a subset of patients from each PMI category for which multiple normal tissues were evaluated (Figure 2). In all patients we noted variability in RIN values among different tissues, ranging from as low as an RIN values of < 2 to > 9 in a single individual (for example, patient RA15-11 in Category III or A164 in Category IV). However, the overall variability was less in PMI Category I samples than for PMI Category IV samples. Thus, RNA quality of one normal tissue retrieved postmortem is not a reliable predictor of RNA quality in a second tissue from that same patient, and good quality samples can be obtained despite the length of the postmortem interval.

**Table 2:** Mean and median RIN values in postmortem tissues^a^

Category	Heart	Kidney	Liver	Lung	Pancreas	Skeletal muscle	Skin	Spleen	Primary	Liver Met	Lung Met
I	7.4 ± 1.9 (7.6)	5.7 ± 1.4 (6.2)	7.3 ± 2.4 (7.6)	7.1 ± 1.7 (6.8)	6.3 ± 2.2 (6.7)	9.3 ± 0.3 (9.3)	8.0 ± 0.5 (7.9)	6.1 ± 2.2 (6.7)	6.6 ± 2.0 (7.2)	6.4 ± 2.7 (6.6)	8.2 ± 1.9 (8.4)
II	6.8 ± 1.7 (6.3)	4.3 ± 2.5 (4.3)	6.2 ± 3.0 (6.7)	5.2 ± 2.2 (5.0)	5.9 ± 1.9 (6.5)	8.6 ± 2.6 (9.5)	6.0 ± 1.6 (6.6)	6.8 ± 2.0 (7.8)	4.5 ± 2.1 (4.5)	5.0 ± 2.9 (3.7)	6.1 ± 2.9 (6.3)
III	5.3 ± 2.4 (5.3)	4.3 ± 1.6 (4.3)	4.7 ± 2.7 (3.3)	6.0 ± 2.5 (7.1)	4.6 ± 2.7 (3.3)	9.2 ± 0.6 (9.4)	6.5 ± 2.2 (6.9)	3.5 ± 1.8 (2.7)	3.6 ± 2.2 (3.2)	3.5 ± 2.3 (2.2)	6.2 ± 3.0 (7.7)
IV	6.8 ± 2.7 (7.9)	3.8 ± 1.9 (2.9)	4.2 ± 3.3 (2.3)	4.3 ± 1.6 (3.7)	3.5 ± 2.8 (2.5)	9.0 ± 0.5 (8.8)	5.0 ± 1.7 (4.9)	4.3 ± 1.5 (4.4)	5.5 ± 2.8 (6.7)	4.7 ± 16 (4.4)	3.4 ± 1.2 (2.8)

a Mean values are expressed as mean ± SD. Median values are shown in brackets.

**Figure 3:**
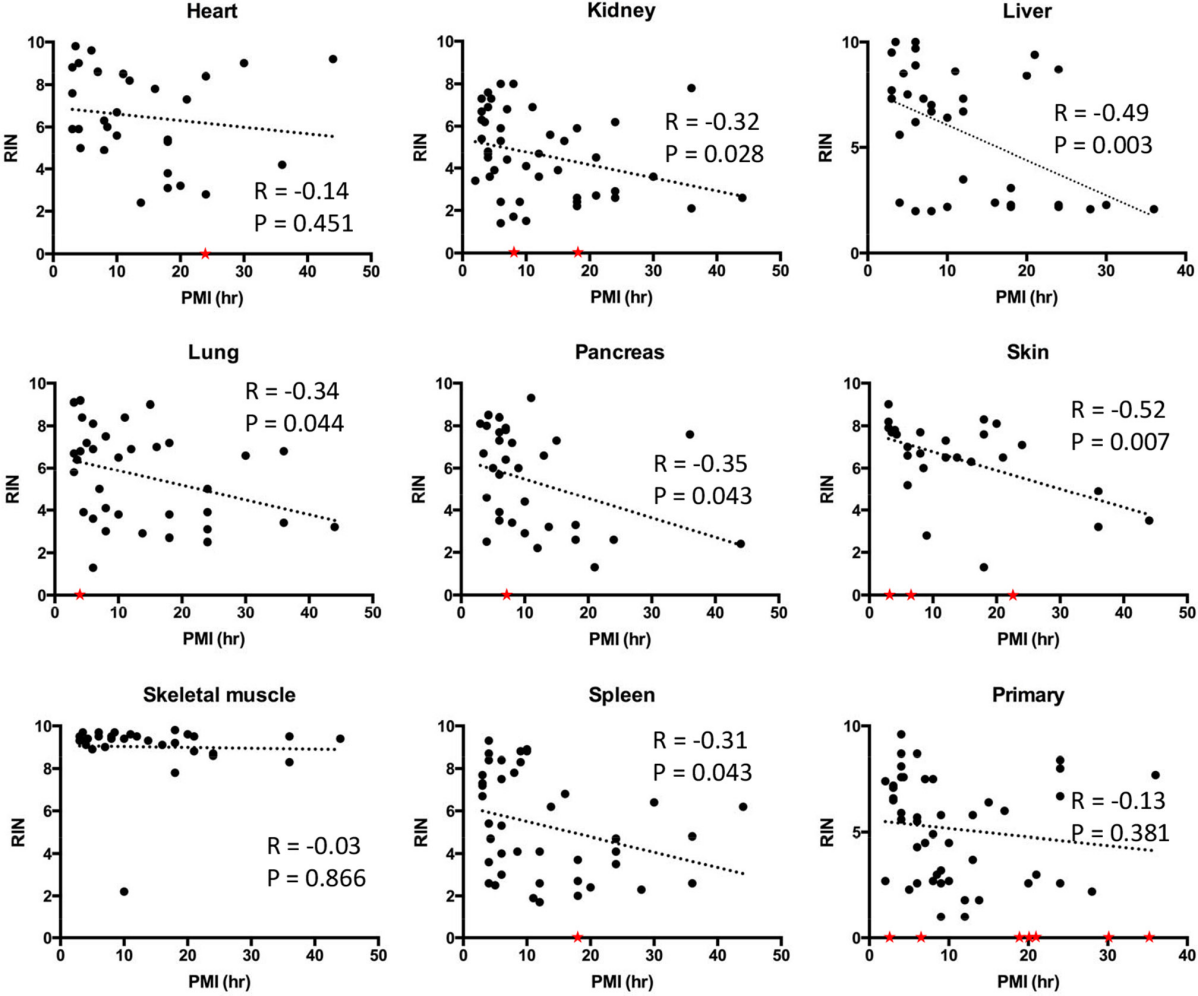
Correlations between RNA integrity numbers and post mortem interval by tissue site Scatter plots were generated by plotting RIN values from each normal tissue type or primary tumors against PMI. Linear regression was performed to create curve fits. Samples with low RNA yield leading to unreported or unreliable RIN were assigned to a RIN value of 0 (red stars) and excluded from linear regression analysis.

**Figure 4:**
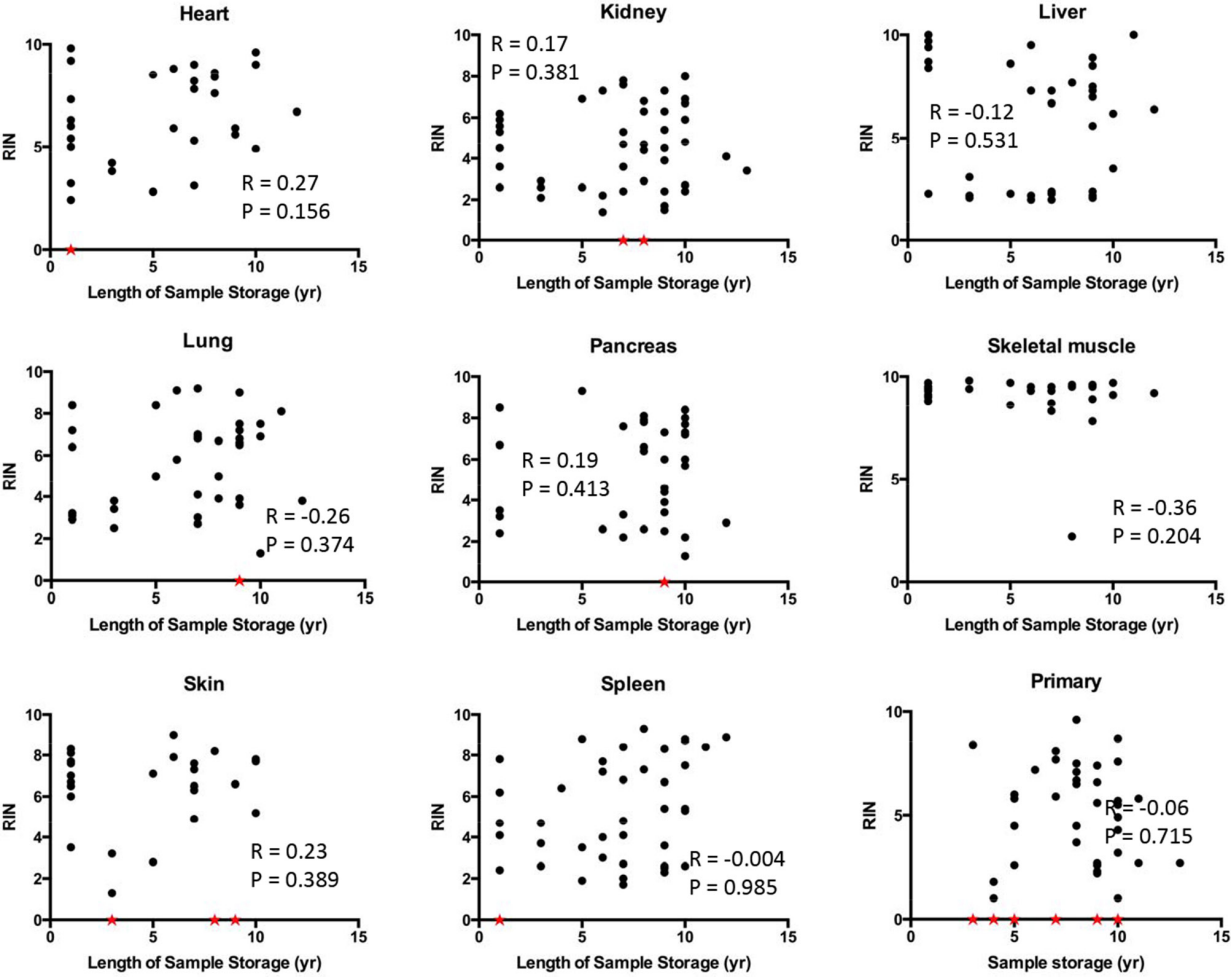
Correlation between RIN and sample storage time Scatter plots were generated by plotting RIN values from each tissue types against sample storage time. Samples with low RNA yield leading to unreported or unreliable RIN were assigned to a RIN value of 0 and indicated by a red star.

We then determined the relationship of RIN values to PMI interval in greater detail by performing correlation analyses. Statistically significant negative correlations were noted for the liver, lung, kidney, pancreas, spleen and skin (Figure 3). Liver and skin showed particularly strong negative correlations between RIN value and PMI with *r* values close to −0.5 and *p value*s < 0.01. By contrast, no correlations were found for the heart or skeletal muscle with the RNA showing remarkable stability and quality in patients with PMIs as long as 36 hours or greater. No correlation was noticed between RIN and the length of sample storage in any of the tissue types examined (Figure 4). Taken together, these results indicate that the RNA quality of normal tissues declines with the elongation of PMI but not storage time, and the extent of degradation is tissue-type specific.

These results nonetheless demonstrate a clear negative correlation between RNA integrity and PMI in most tissue types indicating the importance of this variable. This correlation was established in the presence of several unavoidable confounding factors such as pyrexia, cachexia or prolonged hypoxia in the perimortem period, indirectly confirming that these factors may not be as influential as PMI in predicting RNA quality. We also observed a striking *lack* of correlation of PMI with RIN in normal skeletal muscle, and to a similar extent the heart, supporting the tissue type-specific nature of RNA degradation. In forensic settings, RNA has been shown to be stable in muscle up to 1week after death [18]. While not addressed in this study, tissue-type specific RNA degradation has also been reported in ocular tissues with avascular structures having better RNA quality than vascularized structures such as the ciliary body [23]. Consistent with this notion, we found that normal kidney and liver, two highly vascularized organs, had among the lowest RIN values in each PMI Category. It may be reasonable to speculate that, when controlling for other factors, vascularized tissues are more sensitive to nutrient and oxygen deprivation resulting in a greater extent of sample degradation postmortem. However, given RNA decay is a precisely controlled process in living cells [24], such a process may also contribute to RNA quality in the postmortem period as suggested by Romero et al. [22].

### RNA integrity in cancer tissues

We next wondered if the integrity of ribonucleic acids in cancer tissues parallels that of normal tissues. To address this question, we first analyzed RNA quality in 52 primary tumor tissues and 32 metastatic tumor tissues from the liver and lung. When stratified by PMI Category there were 15 primary tumors and 10 metastases in PMI category I, 18 primary tumors and 10 metastases in PMI category II, nine primary tumors and six metastases in PMI category III, and 10 primary tumors and six metastases in PMI category IV (Table 1). The mean RIN value in primary tumors was 5.16 ± 2.4, and for liver and lung metastases was 5.07 ± 2.51and 6.29 ± 2.72, respectively (Table 2). There was no correlation between RIN values and PMI in primary tumor tissues (Figure 3).

**Figure 5:**
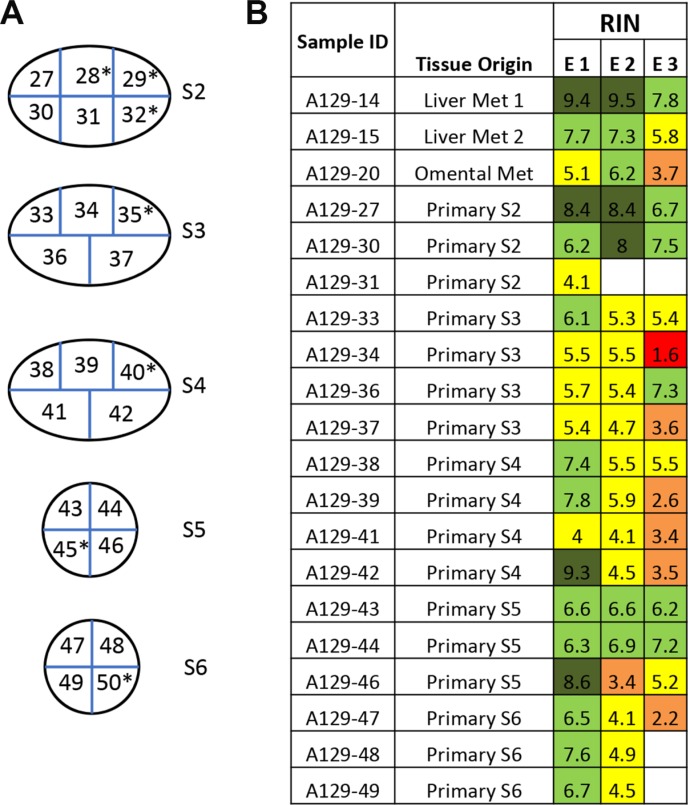
Variation of RNA integrity in primary tumors and metastases in a metastatic cancer case A129 (**A**) One primary pancreatic tumor was bread-loafed as indicated by primary tumor section S2 to S6. Each slice was further cut into equally sized small pieces (approximately 1 × 1 cm) and numbered accordingly to generate sample IDs. Samples with high tumor cellularity were used for RNA extraction and determination of RNA integrity. Samples indicated by an asterisk (*) were excluded due to low tumor cellularity or necrosis. (**B**) Each piece of tissue was trisected and RNA extracted (E1,E2,E3). The color code for RIN value is the same as in Figure 1.

**Figure 6:**
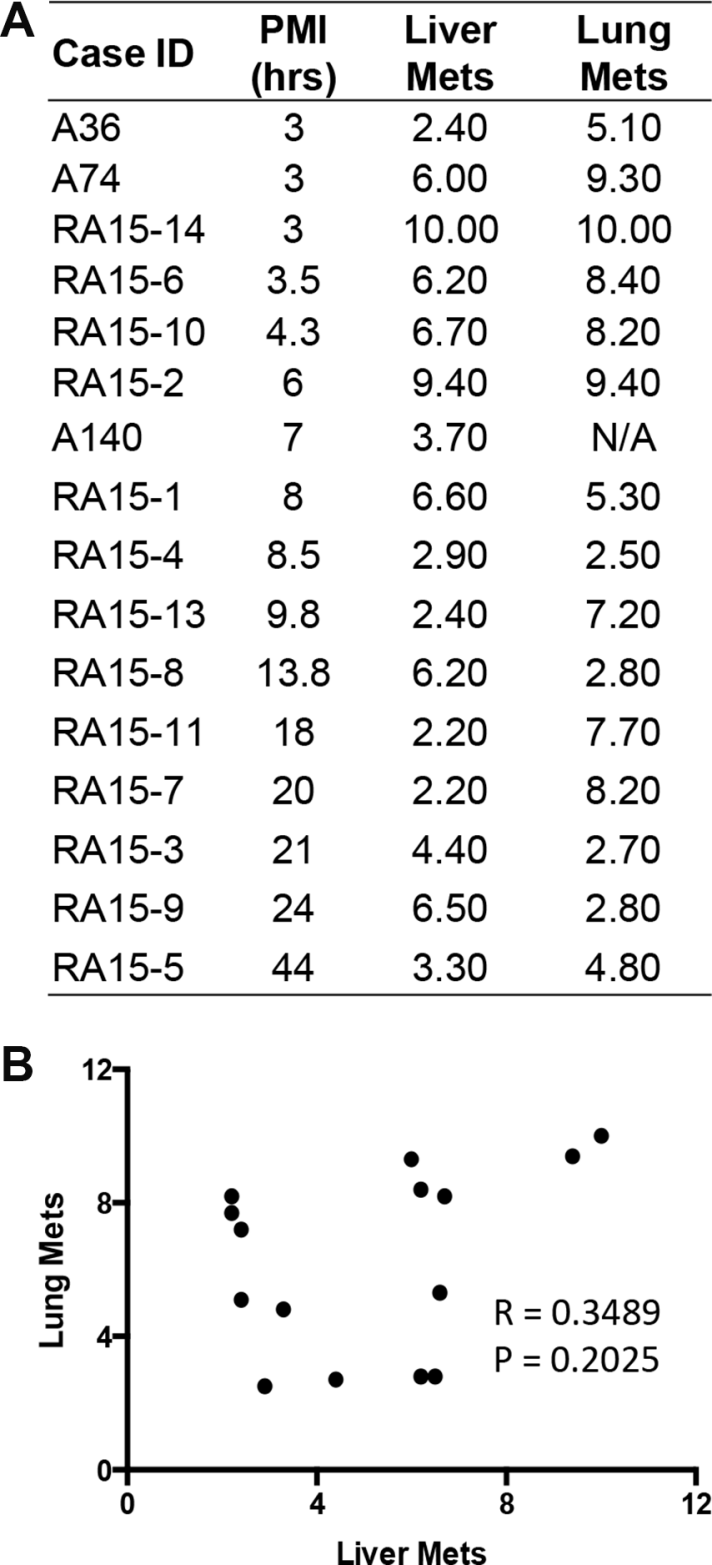
RNA integrity in patient-matched liver metastases and lung metastases (**A**) Numbers listed are the corresponding RIN value for each individual tissue. N/A, data not available due to low RNA yield. (**B**) Scatter plot was generated by plotting RIN values from liver metastases against that from lung metastases. Pearson r and *p value*s were from correlation analysis.

Forty-three of the 52 primary tumors (83%) analyzed were pancreatic ductal adenocarcinomas (PDA) (Figure 1), providing an opportunity to compare the RIN values in primary PDAs specifically to that of normal pancreatic tissues. The mean RNA integrity in PDA tissues was not significantly different from that of normal pancreas tissues when considering all samples (mean RIN 5.4 ± 2.4 versus 5.26 ± 2.56 respectively, *p* = NS), or when limiting the comparison to 17 matched pairs of normal pancreas and primary PDA (mean 6.16 ± 1.96 vs 5.17 ± 2.35 respectively, *p* = NS). This finding thus does not support the long-held “myth” that PDA tissues have worse quality than other tumor types. This may be partially explained by observations that PDA is characterized by a prominent desmoplastic/stromal reaction that is hypovascular compared to adjacent normal pancreas [25]. Nonetheless, we have found that screening multiple geographically distinct samples from different regions of the same neoplasm may be necessary to identify regions with preserved RNA quality, as we have recently found in ongoing work in our laboratory (Figure 5). Finally, we next explored the relationships of metastatic tumor RNA quality between matched liver and lung metastases, i.e. from the same patient. There was no statistically significant correlation (Figure 6), indicating that RNA quality is highly variable among metastases, even within the same patient. Thus, unlike normal tissues that show fairly predictable and tissue-specific degradation in relation to PMI, cancer tissues derived from different organ sites appear less predictable with respect to RNA quality than that of normal tissues.

### DNA integrity in normal tissues is tissue-type specific

While generally more stable than RNA, DNA is also subject to degradation in the postmortem period [18]. Most methods to assess DNA degradation depend on examining selected target(s) to represent the overall sample quality with PCR-based methods among the most popular approach for this purpose [18, 26]. However, the extent to which such methods are reproducible or subject to inter-experiment variation is unknown, as is the extent to which genomic DNA in postmortem tissues follows similar kinetics as RNA.

With these factors in mind we developed a semi-quantitative method to evaluate DNA quality to facilitate comparison among samples from independent experiments. In addition, unlike most studies detecting one locus, we simultaneously examined five chromosome loci of varying potential stability and susceptibility to DNA damage-inducing factors thereby achieving high sensitivity in detecting DNA damage in well-preserved samples (Table 3). DNA was extracted from 36 frozen autopsy samples that were collected from five patients in PMI Category I and five in PMI category IV (Table 4). To facilitate comparisons between RNA and DNA quality, samples with a wide spectrum of RIN values were selected from each PMI category, ranging from as low as 2.3 to as high as 9.4. These included samples from normal liver (*n* = 7), normal kidney (*n* = 9), primary tumors (*n* = 10), liver metastases (*n* = 5) and lung (*n* = 5) metastases.

**Table 3:** Primers for DNA quality analysis and locus information

Locus	UniSTS Number	Primers	Sequence	Size	Locus	Overlapped gene
Locus 1	SHGC105883	F	5′-CCTGGCAAGTAATGGACAATGA-3′	980 bp	Chr13 q14.3	ATP7B
		R	5′-GCCTTTCCAGAGAACTGCAGAC-3′			
Locus 2	STB39J12.SP6	F	5′-TTTCTAGAGCAGTGCAGAGTACTA GGAT-3′	640 bp	Chr4 p15.33	/
		R	5′-TCTTTCCCTCTACAACCCTCTAACC-3′			
Locus 3	STSG50529	F	5′-TGAACAAGGGTTCCAGGATG-3′	560 bp	Chr22 q13.32	/
		R	5′-GAGGTGGGCTTGACTTCGAG-3′			
Locus 4	SHGC147491	F	5′-GGTAAACACACAATGGCCCAG-3′	474 bp	Chr12 q13.13	/
		R	5′-AAAAACGGAAGAAGTCTCTTGGC-3′			
Control Locus	CSNPHARP	F	5′-CATGGCTCACTGGCTTACAA-3′	196 bp	Chr2 q35	SMARCAL1
		R	5′-TTGCCTCTTACAGAGGAGCAG-3′			

**Table 4:** Selected cases for DNA quality analyses^a^

Case ID	PMl (hrs)	Normal Kidney	Normal Liver	Primary Tumor	Liver Metastasis	Lung Metastasis
A36	3	6.7		N/A	2.4	5.1
A74	3	6.3		7.4	6	9.3
A70	4	4.5	5.6*	5.6		
RA15-10	4.3	3.6		7.6	6.7	8.2
A68	5	3.9	7.5	2.3		
RA15-3	21	4.5	9.4	3	4.4*	2.7
A138	24	2.6	2.3	2.6		
RA15-9	24	6.2	8.7	8	6.5	2.8
A73	28		2.1*	2.2		
A165	> 24	2.1	2.1*	8.4		

a Numbers listed under each tissue type are the corresponding RIN value. Samples with DNA damage are in bold and marked with asterisks.

**Figure 7:**
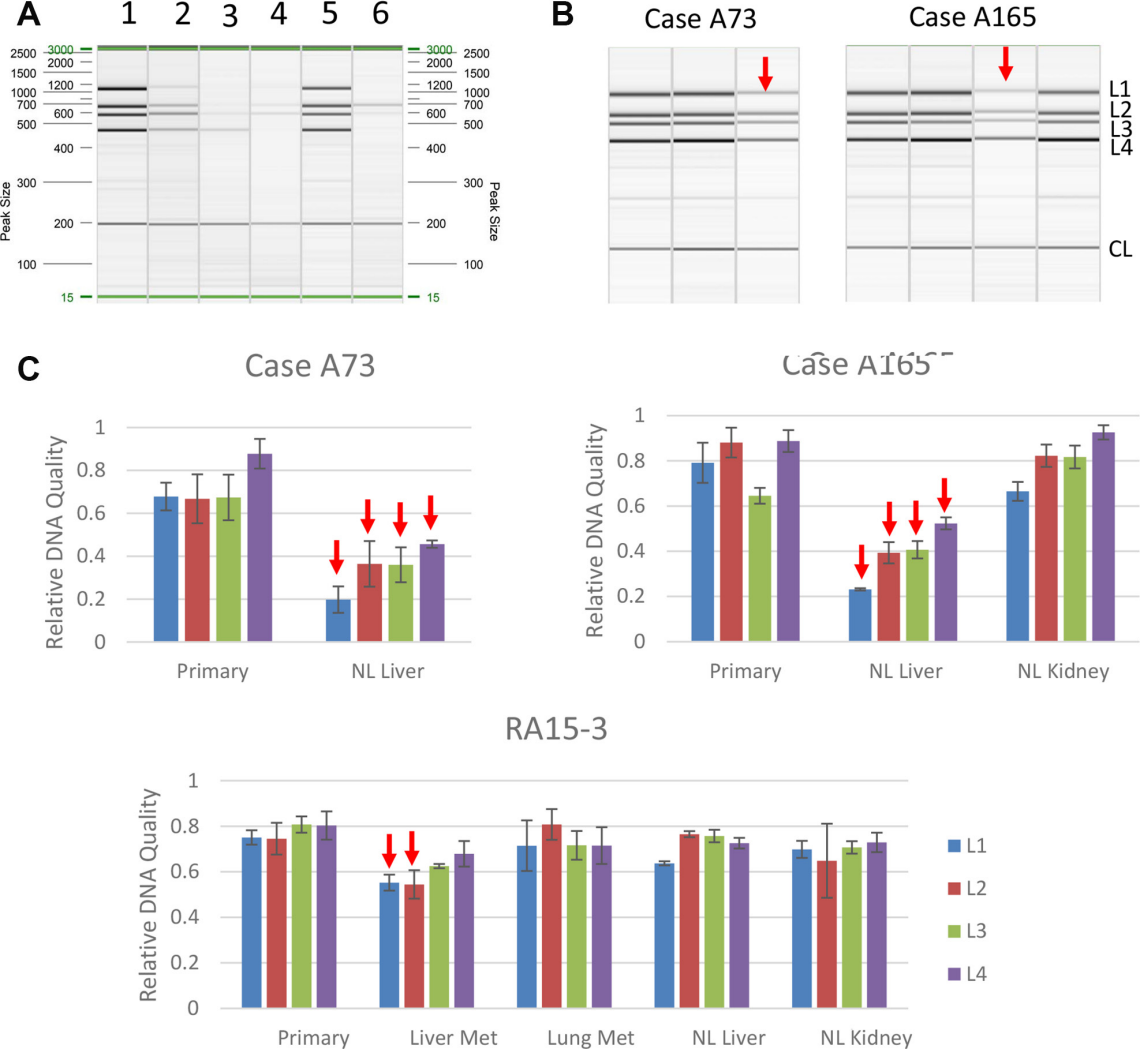
Detection of DNA damage in autopsy samples (**A**) PCR band pattern using control DNA. Lane 1, Intact genomic DNA; Lane 2–4, Genomic DNA sonicated to 500 bp, 300 bp or 150 bp; Lane 5, DNA from a representative frozen autopsy tissue; Lane 6, DNA from a representative formalin-fixed paraffin-embedded (FFPE) tissue sample. (**B**) Representative PCR band patterns of samples collected from two autopsy cases. In each panel, the first lane is intact genomic DNA and the remaiing lanes correspond to the tissue types shown in (**C**) Arrows indicate samples with DNA damage. L1, L2, L3, L4 and CL represent locus 1, 2, 3, 4 and control locus, respectively. (C) Relative DNA quality calculated as described in Materials and Methods. Met, metastasis; NL, normal.

We successfully extracted genomic DNA from all 36 samples including one that failed in RNA extraction. No DNA damage was detected by our assay in 32/36 samples analyzed (89%) even though 17 of these 32 (53%) had RIN values less than 5. In the four samples with DNA damage three were from PMI category IV, two of which showed degradation at all four sensor loci (Figure 7). The remaining two samples showed only moderate damage as reflected by only two of the four chromosome loci affected. All four samples with DNA damage had concurrent RNA degradation (Table 4). Interestingly all four samples with DNA damage were from the liver, three of which were histologically normal, further supporting the greater susceptibility of vascularized tissues to postmortem degradation. All other samples except for these four patients did not show DNA damage, including all 10 primary tumors analyzed. Thus, while DNA damage in postmortem tissues may be an indicator of RNA quality the converse is not true. Moreover, in addition to PMI the kinetics of postmortem DNA degradation may also have tissue type-specific factors.

### RNA sequencing using postmortem RNA samples

Genomic DNA from postmortem tissues has been used successfully for next generation sequencing despite potential DNA degradation [14, 15, 27]. However, given that RNA is more sensitive than DNA during the postmortem interval, its utility in downstream sequencing applications is unknown. As a proof of principle study, we therefore performed RNA sequencing on five matched pairs of normal pancreas and pancreatic cancer tissues, all with an RIN value of 5 or greater.

**Figure 8:**
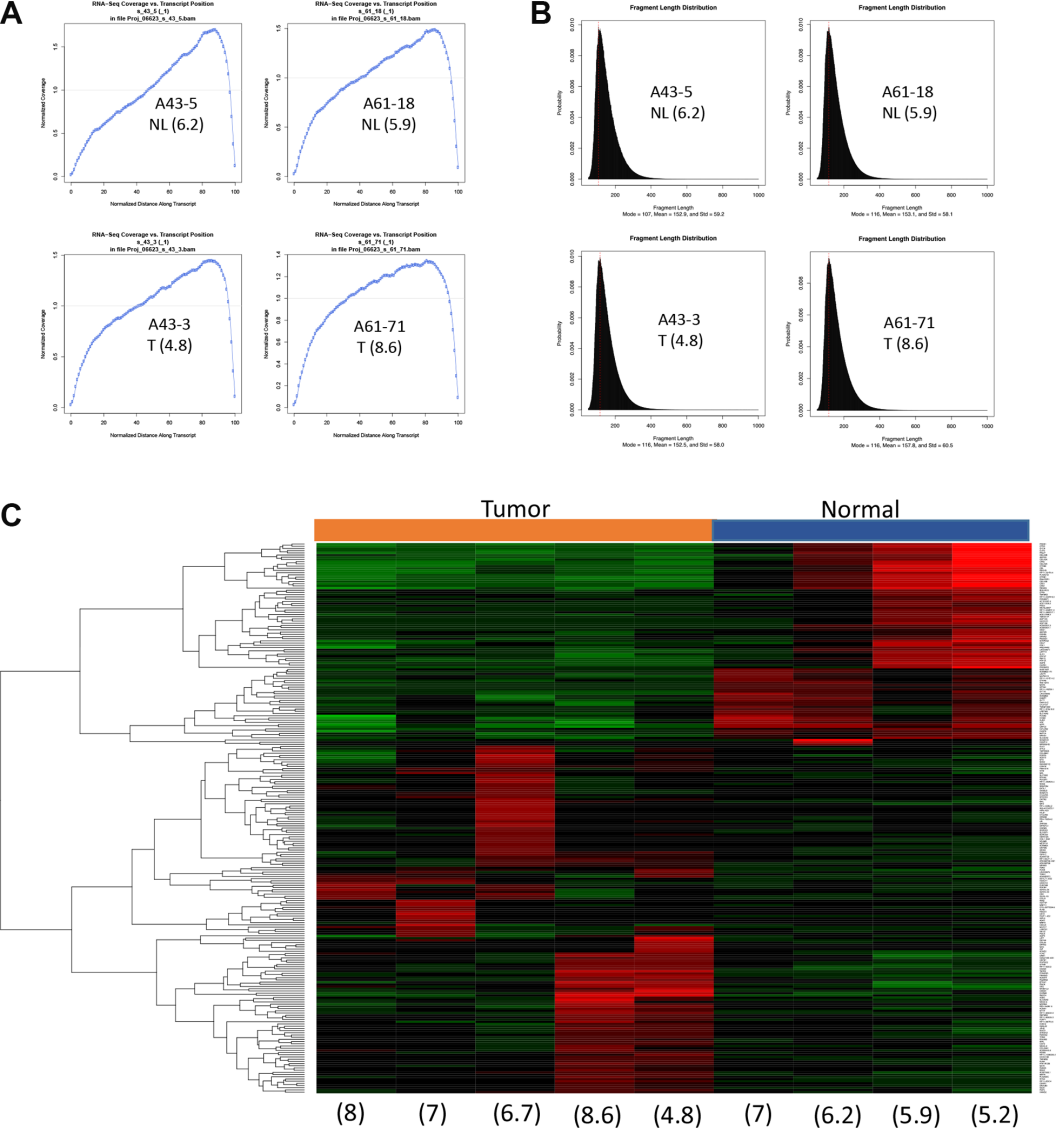
RNAseq clearly separates a tumor signature from the normal counterpart (**A**) Normalized RNA-seq coverage is plotted against transcript position. (**B**) Fragment length distribution. Representative samples with wide spectrum of RIN are shown in A and B. (**C**) Heat map shows expression profile of top 250 differentially expressed genes. Numbers in parenthesis indicate RIN values. Tumor, tissues from primary pancreatic cancer. Normal, tissue from normal pancreas.

Sequencing libraries were successfully generated from all samples using the poly-A enrichment method and used to generate 80 million reads per sample. Moreover, quality metrics of each library showed a sound distribution of coverage along transcripts and fragment lengths irrespective of RIN values (Figure 8A and 8B). One normal sample (patient A105), while showing good quality sequencing data, was excluded because the resulting data indicated contamination by cancer cells. This was confirmed histologically. A heat map of the top 250 genes differentially expressed between five pancreatic cancers and the remaining four normal pancreata showed a pattern that clearly discriminated the two groups (Figure 8C). Genes transcripts overrepresented in normal tissues included PRSS1 (cationic trypsinogen), CPA2 (pancreatic specific carboxypeptidase), AMY2A (pancreatic amylase 2), and the pancreatic developmental transcription factor PTF1A consistent with the greater abundance of acinar cells or cells with stem-like properties within these samples [28–30]. Cancer samples showed greater heterogeneity with respect to the most differentially expressed genes. Genes overrepresented in the cancer samples included PSCA [31], MMP3 [32], MMP11 [33] and SOX2 [34]. The small sample size otherwise precluded a more thorough classification of each carcinoma's subtype as recently described [11]. Finally, we leveraged our sequencing coverage to identify potential fusion events. We identified two recurrent fusion events, *AXGP1-GJC3* and *SIDT2-TAGLN,* in six of nine postmortem RNA samples (three normal and three tumor, including two normal-tumor matched pairs). These two fusions were recently reported in normal pancreatic tissues within the context of a larger pan-tissue analysis [35].

Collectively, these data are encouraging and suggest that despite being collected under postmortem conditions RNA samples can provide biologically meaningful information in downstream analyses. These findings are particularly exciting considering recent reports of improved methods to directly assess mRNA integrity and control for it in analyses of RNAseq data [22, 36, 37]. While we did not utilize these methods for our pilot comparison, we nonetheless were able to discern gene expression signatures of known biologic importance in normal and pancreatic cancer tissues. With larger sample sizes and use of these methods it is reasonable to expect novel observations to be made in cancer tissues, for example with respect to treatment resistance or subclonal evolution. It is also important to note that our data do not indicate the RIN threshold value below which RNA sequencing cannot be performed, and in light of the pilot data shown it may be worth studying samples with low RIN values as well particularly as 39% of our samples had an RIN value of < 5.

Previous evaluations of the quality of biomolecules in postmortem tissues have been in the context of brain banking, forensic analyses or minimally invasive autopsies [19, 38–40]. However, such an analysis using tissues obtained from a research autopsy program for cancer research has not been performed. Such information is critical to know with the growing interest in creation of biobanks from postmortem tissues of cancer patients, and use of these materials for ongoing scientific discovery and collaborations. While the scope of our dataset parallels that of another recent study [41], it differs in that we also studied DNA integrity as well and cancer tissues derived from different primary or metastatic sites. Nonetheless, our data is consistent with these prior studies that indicate nucleic acid quality of normal tissues is affected by a considerable number of factors in addition to the postmortem interval. At least one of these is likely the cause of death as we noticed several patients with a short PMI (< 3 hours) with exceedingly poor RNA quality, all of whom died of sepsis (personal observations, C.I.D.).

Despite an increasing demand for research autopsy samples in the cancer research community, collecting high quality tissue samples is challenging due to numerous factors that can be legal, ethnic, emotional or social in nature. Exclusion of samples with a degree of poor quality is not always realistic, particularly when not all downstream applications are equally sensitive to sample quality. Thus, analyses that can incorporate these variables would be expected to improve comparisons across patients, tissue types and sample sets. Table 5 summarizes these variables and their effects on RNA quality. Our hope is that these findings provide insight on the sample variability expected from research autopsy resources and ultimately facilitate data interpretation.

**Table 5:** Considerations when performing RNA sequencing from postmortem tissues

Factor^a^	Examples	Potential Effect on RNA Yield, Quality or Data^a^
*Ultimate Cause of Death of Patient*	Sepsis, Prolonged Hypotension	Lower RNA yield and quality of the affected tissues
	Acute Myocardial Infarction, Stroke	Minimal effect
*Postmortem Interval*	Prolonged	Lower RNA yield and quality that is tissue type dependent
*Postmortem Refrigeration*	Immediate delivery of patient to morgue after death	Can negate effects of prolonged postmortem interval
*Tissue type*	Normal tissue	RNA yields and quality are tissue type dependent and related to cause of death and PMI
	Cancer tissue	RNA yields and quality may not be tissue type dependent and related to extent of necrosis or autolysis in the sample
*Tissue storage*	Prolonged storage (e.g. >10 years)	Lower RNA yields
*Tissue handling*	Multiple freeze/thaw cycles	Lower RNA quality
	Prolongation between death and tissue processing	Lower RNA quality
	Prolongation between time of processing of different samples during autopsy	Potential intrapatient variability in RNA quality
*RNA handling*	Multiple freeze/thaw cycles	Lower RIN values
*Method of RNA Extraction*	RNeasy mini plus kit	RNA > 200 bp
*Method of Library Preparation*	PolyA enrichment (requires relatively high quality RNA (RIN > 5)	Higher transcriptome coverage
	Ribosomal depletion (best for degraded RNA)	Lower transcriptome coverage

a These potential effects are based on the assumption that all other factors for the patient and tissues are optimal. Should more than one adverse factor exist an even greater loss of RNA yield or quality could be expected.

## MATERIALS AND METHODS

### Tissues

Autopsy samples were collected in association with the Johns Hopkins Gastrointestinal Cancer Rapid Medical Donation Program (GICRMDP) or the Memorial Sloan Kettering Cancer Center Medical Donation Program (MDP). Both programs were approved by the IRB at their respective institutions and in accordance with an assurance filed with and approved by the U.S. Department of Health and Human Services. Details of the program have been described previously [42]. Briefly, the tissue harvesting protocol consists of opening of the body cavity using standard techniques and sterile sampling of a variety of normal tissues, the primary tumor if present and each grossly identified metastasis using a sterile blade and forceps. For snap-freezing, tissues were collected in 1.7 ml cryovials and immediately placed in liquid nitrogen before transferring to −80°C for long-term storage. Information regarding patient characteristics were recorded including the postmortem interval (PMI), defined as the time from death to the time of first incision. In all instances the time from the start to end of tissue sampling was ≤ 2 hours.

### RNA extractions

For each sample, approximately 30 mg tissue was carefully harvested on ice and homogenized using the Fastprep-24 system with ceramic beads (MP Biomedicals). Total RNA was then extracted using RNeasy mini kits or Fibrous tissue mini kits (Qiagen) following the manufacturer's instruction. The RNA extraction step was carefully monitored by simultaneously extracting RNA from freshly sacrificed snap frozen mouse tissues of the same tissue type as a positive control. Total RNA was eluted in DNase/RNase free water. RNA was quantified using a Nanodrop 2000 spectrophotometer (Thermo Fisher Scientific) and subsequently stored at −80°C. No more than 10 samples were extracted at one time. To confirm reproducibility of our extraction procedure, 10 samples were randomly selected and RNA re-extracted from the same tissue. In all cases similar yields were obtained from the first and second extraction. Any sample for which RNA could not be extracted was independently extracted at least one more time to rule out technical errors during the extraction procedure.

### RNA integrity analysis

The RNA integrity number (RIN) was determined using an Agilent 2100 Bioanalyzer (Agilent Technologies) with an RNA nano-kit system as described in the manufacturer's instructions. For each chip analysis, a commercially available tissue-matched control human RNA with high RNA quality (ZYAGEN) was run together with autopsy RNA samples to monitor the whole analysis procedure. Data reproducibility was confirmed by repeating the RNA chip analysis in 10 randomly selected samples at least two days apart from the first analysis. In all cases the results were highly reproducible with an overall difference in RIN value between the two chip assays of < 1.

### Genomic DNA extractions and PCR amplification

Genomic DNA was extracted using DNeasy Mini Kits (Qiagen) according to the manufacturer's instructions. Genomic DNA was quantified by Qubit fluorometer (Invitrogen) and diluted to 20 ng/ul. 20 ng diluted DNA was subjected to PCR amplification in a total volume of 20 ul. PCR was carried out using a *Taq* PCR Core Kit (Qiagen). PCR conditions were 94°C for 2 min; 35 cycles of 94°C for 1 min, 60°C for 1 min and 72°C for 1 min 30 sec followed by 1 cycle of 72°C for 7 min. 3 ul of PCR products amplified from each locus were pooled in a new PCR tube and run on DNA screening gel cartridges (Cartridge ID C15D4D3A11) on a QIAxcel advanced system (Qiagen) according to manufacturer instructions.

### DNA quality analysis using two-step data normalization

We adapted the qualitative multiplex PCR assay developed by Sigma-Aldrich to detect DNA damage in formalin-fixed paraffin embedded (FFPE) tissues (http://www.sigmaaldrich.com/technical-documents/articles/life-science-innovations/qualitative-multiplex.html) for analysis of snap frozen postmortem tissues. The original assay consists of five primer sets derived from the NCBI UniSTS database that amplify products ranging from 132 bp to 295 bp; some or all of these products will fail to amplify when DNA damage is present. PCR primers were modified to increase the amplicon sizes from 474 to 980 bp (Table 3). One loci, a 196 bp amplicon, was used as an internal control to normalize PCR template input and amplification efficiency. The remaining four amplicons are located within known chromosome fragile sites that are relatively more susceptible to hydrolytic DNA damage and therefore serve as sensors of DNA quality [43]. To avoid amplification bias that may be introduced during multiplex PCR each loci was amplified independently and then pooled for visualization and band quantification as described above. Commercially available human genomic DNA was used as an intact control and sonication fragmented DNA as damaged DNA control. Intact and fragmented DNA controls were analyzed in parallel with all human samples.

Data were analyzed using a two-step data normalization to acquire a relative DNA quality of each sensor loci amplicon compared to the control amplicon. First, for each sample the band Intensity from each of the four sensor loci were normalized to that of the control locus. A standard band intensity was established from the intact control DNA by using mean values from three independent amplifications. Second, the relative band intensity from all samples was subsequently normalized to the standard. Based on this metric samples with perfect DNA quality have a value of 1 and samples with complete DNA degradation will have a value of 0. Values below the threshold of 0.6 were arbitrarily defined as having DNA damage.

### RNA sequencing

Selected RNA samples from postmortem tissues with RIN value above 5 were used for RNA sequencing. RNA sequencing was performed in the MSK Genomics Core using the Illumina Truseq RNA sample prep protocol. Briefly, RNA sequencing libraries were generated with poly-A-enrichment method and sequencing was performed on an Illumina HiSeq2000 following standard protocols. Reads were paired-end 50 bp in length with a total of 80 millions of reads per sample. All sequenced libraries were mapped to the human genome (hg19) using rnaSTAR aligner [44]. After mapping the expression count matrix was computed from the mapped reads using HTSeq (www-huber.embl.de/users/anders/HTSeq). The raw count matrix generated by HTSeq was then processed using the R/Bioconductor package DESeq (www-huber.embl.de/users/anders/DESeq) [45], which is used to both normalize counts and to identify differentially expressed genes between two conditions. A gene was declared differentially expressed if the fold-change was greater than 2 and the adjusted *p-value* was less than 0.05. Normalized counts were log2 transformed after addition of 1 to all values. Hierarchical clustering was performed using the R hclust function with the Euclidean distance measure on normalized log2 transformed counts after addition of 1 to all values. A heatmap was generated using the heatmap. 2 function from the gplots R package. The data plot shows the mean centered normalized log2 expression of the top 250 genes differentially expressed between tumor and normal tissues. To to detect fusion chimeras from RNA-seq data, meta-analysis that runs four fusion detection algorithms (ChimeraScan, FusionCatcher, MapSplice and DeFuse) was applied. The pipeline computes a meta-score for each detected fusion thus alleviating a problem of high numbers of false positives in each method taken independently.

### Statistics

Statistical analysis was performed using GraphPad Prism Version 6.0 (GraphPad Software, Inc. La Jolla, CA). To determine the relationship between RIN and PMI, correlations were performed to determine the *R*^2^ value and *p value*. Curve fits were added to scatterplots by performing linear regressions. Patient-matched tumor-metastasis comparisons were compared by a two-tailed Student *t* test. A *p value* of 0.05 or less was considered statistically significant.
